# Loneliness trajectories and dementia risk: Insights from the HUNT cohort study

**DOI:** 10.1002/dad2.70154

**Published:** 2025-07-29

**Authors:** Ragnhild Holmberg Aunsmo, Bjørn Heine Strand, Sverre Bergh, Thomas Hansen, Mika Kivimäki, Sebastian Köhler, Steinar Krokstad, Ellen M. Langballe, Gill Livingston, Fiona E. Matthews, Geir Selbæk

**Affiliations:** ^1^ Norwegian National Centre for Ageing and Health Vestfold Hospital Trust Tønsberg Norway; ^2^ Faculty of Medicine Institute of Clinical Medicine University of Oslo Oslo Norway; ^3^ Verdal municipality Verdal Norway; ^4^ Department of Geriatric Medicine Oslo University Hospital Oslo Norway; ^5^ Physical Health and Ageing Norwegian Institute of Public Health Oslo Norway; ^6^ Research Centre for Age‐related Functional Decline and Disease Innlandet Hospital Trust Ottestad Norway; ^7^ Department of Epidemiology and Public Health University College London London UK; ^8^ Department of Psychology Finnish Institute of Occupational Health Helsinki Finland; ^9^ Alzheimer Center Limburg, Dept Psychiatry & Neuropsychology Maastricht University Maastricht The Netherlands; ^10^ Institute for Mental Health and Neuroscience Maastricht University Maastricht The Netherlands; ^11^ HUNT Research Centre, Department of Public Health and Nursing Norwegian University of Science and Technology Levanger Norway; ^12^ Department of Public Health and Nursing University of Science and Technology Trondheim Norway; ^13^ Department of Psychiatry Levanger Hospital Nord‐Trøndelag Hospital Trust Levanger Norway; ^14^ Division of Psychiatry University College London London UK; ^15^ Institute for Clinical and Applied Health Research University of Hull Hull UK

**Keywords:** course of loneliness, dementia, loneliness, loneliness and dementia, long‐term loneliness, risk factor, risk of dementia, trajectories of loneliness

## Abstract

**INTRODUCTION:**

Loneliness is postulated to be a risk factor for dementia. However, the findings are inconsistent, and long‐term studies on this association remain scarce.

**METHODS:**

In all, 9389 participants self‐reported loneliness in the Trøndelag Health Study (HUNT) in HUNT1 (1984–1986), HUNT2 (1995–1997), and/or HUNT3 (2006–2008) and underwent cognitive assessment in HUNT4 (2017–2019) at age 70 years or older. Logistic regression was employed to analyze the association between the course of loneliness and dementia, with those never lonely as a reference.

**RESULTS:**

In the fully adjusted model, the odds ratio (OR) for persistent loneliness was 1.47 (95% confidence interval [CI] 1.10, 1.95). This attenuated when adjusting for depression (OR 1.28, 95% CI 0.95, 1.72).

**DISCUSSION:**

Persistent loneliness from midlife into older age, as well as becoming lonely, were associated with increased odds of dementia, whereas transient loneliness in midlife was not. These findings underscore the importance of reducing loneliness.

**Clinical trial registration:**

The study was registered with ClinicalTrials.gov (NCT04786561) and is available online .

**Highlights:**

- Persistent and incident loneliness was associated with a higher risk of dementia.
- Transient loneliness was not associated with a higher risk of dementia.
- Loneliness 11 years before to the cognitive assessment was associated with dementia.
- Reducing the sense of loneliness might reduce or delay the onset of dementia.

## 1 BACKGROUND

Due to population ageing, dementia is an increasingly significant societal challenge. Its worldwide prevalence is estimated to rise from 57.4 million in 2019 to approximately 152.7 million people in 2050,^1^ and in Norway, the corresponding numbers are 101,000 in 2019 and 237,000 by 2050.^2^ The Lancet Commission on Dementia Prevention, Intervention, and Care has estimated that 14 modifiable risk factors—including lack of social contact—account for 45% of dementias.^3^ A frequent problem among older adults is loneliness, understood as a subjective, unpleasant experience which occurs when a person's network of social relationships is deficient in quality.^4^ It has been estimated that worldwide, nearly three in 10 older adults experience loneliness,^5^ although the prevalence varies among groups and is higher in specific communities and impaired individuals.^6^ A recent umbrella review and Delphi study suggested incorporating social contact, hearing impairment, and sleep into the existing dementia risk score (“LIfestyle for BRAin health” [LIBRA] score);^7^ they also found that functional operationalization's—such as loneliness and social engagement—are more strongly associated with dementia risk than structural operationalization's—such as social network size and living alone. The Delphi study team called for more long‐term cohort studies.

Although reviews have found association between positive concepts of social participation and reduced dementia risk, and between loneliness and increased risk, the longitudinal evidence supporting this is mixed—possibly due to inconsistent use of scales measuring loneliness, reverse causation bias, confounding, and differences in the follow‐up time.^8^ One meta‐analysis showed a stronger association over longer follow‐ups^9^; however, only a few studies have a follow‐up period exceeding 10 years.^10^, ^11^, ^12^, ^13^, ^14^ Another study defined five trajectories of loneliness in the English Longitudinal Study of Ageing (ELSA) over 8 years and found that those with long‐term and persistently increasing loneliness belonged to a high‐risk group for dementia.^15^ Yet another study, drawing on six waves of the ELSA study separated by 2‐year intervals,^16^ found that loneliness was associated with poorer cognitive function. The role of depression in the association between loneliness and dementia varies across studies,^17^ although one recent study concluded that depressive symptoms significantly mediated the relationship between loneliness and incident dementia, with no mediation observed in the opposite direction.^18^

Existing observational studies in the literature indicates that loneliness in the years preceding reduced cognitive function is associated with dementia. However, studies with extended follow‐ups and repeated assessments of loneliness are scarce^8^; hence, it remains unclear whether increased loneliness is an effect (rather than a cause) of dementia, what the impact of single versus repeated episodes of loneliness is, and whether the association is independent of co‐occurring depression. Thus, knowledge of the long‐term effect and course of loneliness in relation to dementia risk remains limited. Furthermore, the rates of cognitive decline and stress susceptibility may differ between men and women;^19^, ^20^ emphasizing the need for studies examining sex differences in the association between loneliness and dementia.^21^

Our aim was to examine whether the course of the subjective feeling of loneliness over the three decades preceding a dementia diagnosis was associated with increased dementia risk, as well as to determine whether this association varied by sex or depending on the length of follow‐up period.

**RESEARCH IN CONTEXT**

1. **Systematic review**: We searched PubMed, PsychINFO, and Cinahl for articles published in English from 2015 to November 2024, for studies examining the relationship between aspects of social relations and cognitive health. Previous longitudinal studies have found an increased risk of dementia associated with little social contact and/or loneliness. Few studies have longer than 10 years of follow‐up and thus examined the long‐term consequences of loneliness. Whether transient loneliness enhances the risk of dementia is even less described.
2. **Interpretation**: We analyzed data from four waves over three decades in a large cohort of 9345 participants and found that persistent and incident loneliness were associated with an increased prevalence of later dementia, whereas transient loneliness was not.
3. **Future directions**: Our findings may indicate that adapting or re‐establishing social connections, and thereby reducing the sense of loneliness, also reduces the risk of dementia. Differences between men and women should be further investigated.

## 2 METHODS

### 2.1 Study design and population

**FIGURE 1:**
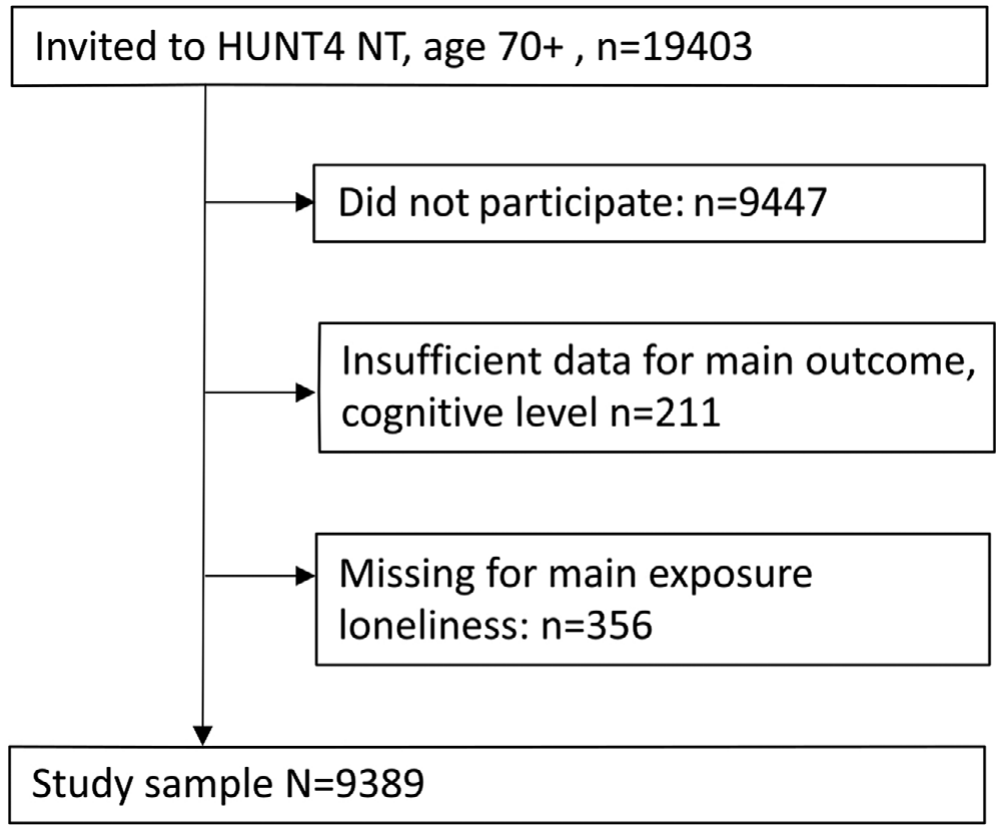
Flow‐chart of study population aged 70+ years in HUNT4 (2017‐^19^) in the HUNT Study (Norway). HUNT, Trøndelag Health Study

Our data come from the population based Trøndelag Health Study (HUNT). At the fourth wave (HUNT4 2017‐2019), those aged 70 years and older (*n* = 19,403) were invited to participate in a comprehensive assessment of cognitive and physical function, and 9930 (51.2%) did so (HUNT4 70+). Of these, 9745 participants provided sufficient information for a conclusive assessment of dementia status. Of these participants, 9389 had participated in at least one of the earlier HUNT waves, namely HUNT1 (1984–1986), HUNT2 (1995–1997), or HUNT3 (2006–2008), and thus were included in our analysis (Figure 1). The HUNT study is further described in the Supplementary Material. Repeated data on loneliness enabled us to classify participants into different courses of loneliness and compare these groups in terms of their risk of dementia at HUNT4.

The HUNT4 70+ assessment included measurements of blood pressure, heart rate, height and weight, and tests of cognitive function, as well as questionnaire surveys on subjective health and occupation, chronic disease, lifestyle, and functioning. The assessment was conducted at a location chosen by the participant, which could be a research field station, their home, or a nursing home.^2^

For the analysis of the association between the course of loneliness and dementia, we used covariates as measured at HUNT3, which was the last point of registration of loneliness, in line with others;^13^ hearing was not measured at HUNT3, and so for this we used results from HUNT2. In the analysis of the association between dementia at HUNT4 and being lonely at separate waves, we used the measures of the covariates from the corresponding wave, with the exception of hearing threshold level (measured only at HUNT2) and income (not available at HUNT1).

### 2.2 Assessment of dementia

Cognitive function was tested using the Montreal Cognitive Assessment (MoCA). For those who scored 22 or more on the MoCA, a more in‐depth test of memory, the Word List Memory Task (WLMT), was used. For participants living in nursing homes with severe cognitive impairment, the eight‐item Severe Impairment Battery was administered instead. The tests are further described in the Supplementary Material. For those who reported subjective cognitive decline or scored below the predefined threshold on the cognitive tests, a caregiver interview was conducted concerning the participant's cognitive impairment, symptom course, neuropsychiatric symptoms, and activities of daily living (ADL) functioning. The dementia diagnosis was determined independently by two clinical and research experts from a diagnostic work‐up group of nine medical doctors who were specialists in geriatric medicine, old‐age psychiatry, or neurology.^2^ The diagnostic criteria of the Diagnostic and Statistical Manual of Mental Disorders, 5th edition (DSM‐5) were applied to determine MCI (mild cognitive impairment) or dementia (major neurocognitive disorder).^22^ The assessment and diagnostic procedures for dementia diagnosis have been further described in previous work.^2^

### 2.3 Assessment of loneliness

Loneliness was measured using a single‐item question asked at each HUNT wave, with responses dichotomized into “lonely” versus “not lonely”. In HUNT1, the question was, “Do you often feel lonely?”, with five response categories: those who responded “very often”, “often”, or “sometimes” were classified as lonely, while those who responded “very rarely” or “never” were classified as not lonely. In HUNT2 and HUNT3 the question was, “In the last two weeks, have you felt lonely?”, with the response options “no”, “a little”, “a good amount”, and “very much”: the last three categories were defined as lonely, while the response option “no” was defined as not lonely. To define courses of loneliness, we combined the responses in the HUNT1–HUNT3 waves and categorized participants into four groups: no loneliness (not lonely at any point of HUNT1‐3); transient loneliness (not lonely at HUNT3, but lonely at HUNT1 and/or HUNT2); incident loneliness (lonely at HUNT3, but not lonely at HUNT1 and/or HUNT2); and persistent loneliness (lonely at all points of HUNT1‐3).

### 2.4 Covariates

We considered confounding and mediating factors to help disentangle the causal pathways from loneliness to dementia: to this end, we constructed a directed acyclic graph based on relevant literature (Figure S1). Sex and age were registered at participation, with age used as a continuous variable. Income (total personal income including tax, divided into quartiles) and education (unspecified/none, primary school, secondary school, university up to 4 years, and university more than 4 years) were obtained from linked records from Statistics Norway. Body height (cm) and weight (kg) were measured at every wave. Body mass index (BMI) was calculated as weight in kilograms divided by height in meters squared (kg/m^2^). Physical activity (inactive versus not inactive), alcohol consumption (frequency of intake), smoking history (current, ever or never), diabetes (yes/no), history of heart attack (yes/no) and apoplexy (yes/no), and depression (defined using the Hospital Anxiety and Depression Scale (HADS)–depression referring to a depression subscale score ≥8) were all self‐reported at each HUNT wave. Hearing level was measured at HUNT2 only. Further descriptions of the covariates are available in the Supplementary Material, including in Table S1.

### 2.5 Ethical approval

The study adhered to the guidelines for the protection of human data concerning safety and privacy at the Norwegian National Centre for Ageing and Health. The present analysis is part of the project “Depression, anxiety and social relationships as risk factors for dementia”, which was approved by the Norwegian Regional Ethics Committee for Medical Research (REC, reference number 182824). All participants gave written informed consent.

### 2.6 Statistical analysis

STATA 18 was used for the statistical analysis. Logistic regression was used to examine the association between the course of loneliness (exposure) and dementia (outcome). A set of models were fitted: first, a model adjusted for age and sex (Model 1): then, a model which additionally included the covariates education, income, hearing threshold level, frequency of alcohol use, obesity (BMI ≥ 30), physical activity, history of smoking, stroke, myocardial infarction, and diabetes (Model 2): and finally a model which additionally included living alone as a covariate (Model 3). To consider the impact of depression, which might be on the causal pathway between loneliness and dementia, depression at HUNT3 was added as a covariate in a subsequent analysis (Model 4). Possible sex differences in these associations were investigated by including the interaction term sex*loneliness; a similar approach was applied to investigate any moderating effects of age group (≤ 80 years vs. > 80 years). Finally, to examine the role of the timing of loneliness in dementia risk, separate regression models were performed for the associations of loneliness at each study wave with dementia at HUNT4. These analyses were performed for both the dichotomized loneliness and the four‐category degree of loneliness. The adjustment variables were the same as in model 3. The analyses were performed using an imputed dataset (described in the following section). As a sensitivity analysis, a complete case analysis was conducted by repeating the main analyses of the course of loneliness and of loneliness at each wave and examining the associations thereof with dementia at HUNT4.

### 2.7 Imputation of missing values

Missing data were handled using multiple imputation by chained equations (MICE), this produced 50 imputed datasets, which is adequate according to recommendations.^23^ Year of birth, sex, and education were used as non‐missing variables in the prediction, while loneliness and all other covariates had missing values, which were imputed. The HADS variables were imputed as recommended in literature, namely by using participant‐specific mean values^24^ before the MICE procedure to reduce the number of variables in MICE. Details of missing data are available in Table S1: briefly, the loneliness variable had 17.3%–20.4% missing, the other variables with the most missing were physical activity (29.2%–52% through HUNT1‐3), alcohol consumption (13.4%–26.9% missing), smoking history (9.4‐19.9% missing), and BMI (7.9%–11% missing).

## 3 RESULTS

The crude prevalences of loneliness were 24.2% at HUNT1, 16.2% at HUNT2, and 18.8% at HUNT3 (Table 1). The corresponding prevalences in the imputed data were 24.9%, 17.4%, and 20.2%, respectively. Women reported loneliness more often than men: at HUNT3, 63% of the participants reporting loneliness were women, compared to 51% of those not reporting feeling lonely. Loneliness was associated with lower education, lower income, living alone, anxiety, depression, obesity, and smoking.

**TABLE 1:** Characteristics of participants by loneliness in the HUNT Study (Norway), at HUNT1 (1984–1986), HUNT2 (1995–1997), and HUNT3 (2017–2019): *N* = 9389

Parameter	Feel lonely at HUNT1		Feel lonely at HUNT2		Feel lonely at HUNT3	
	No	Yes	No	Yes	No	Yes
*N*	5662 (75.8%)	1809 (24.2%)	6506 (83.8%)	1261 (16.2%)	6302 (81.2%)	1463 (18.8%)
	Mean (SD)	Mean (SD)	Mean (SD)	Mean (SD)	Mean (SD)	Mean (SD)
Age at participating	44.9 (6.49)	45.5 (6.94)	56 (6.28)	56.6 (6.98)	66.7 (5.88)	68.1 (6.85)
Hearing TL			4.79 (7.94)	5.98 (9.34)	4.56 (7.66)	6.24 (9.19)
Depressive symptoms					3.05 (2.65)	5.22 (3.66)
Dementia	*N* (%)	*N* (%)	*N* (%)	*N* (%)	*N* (%)	*N* (%)
No dementia	4828 (85.3%)	1469 (81.2%)	5629 (86.5%)	1026 (81.4%)	5581 (88.6%)	1193 (81.5%)
Dementia	834 (14.7%)	340 (18.8%)	877 (13.5%)	235 (18.6%)	721 (11.4%)	270 (18.5%)
Sex						
Women	2968 (52.4%)	1190 (65.8%)	3429 (52.7%)	768 (60.9%)	3257 (51.7%)	919 (62.8%)
Men	2694 (47.6%)	619 (34.2%)	3077 (47.3%)	493 (39.1%)	3045 (48.3%)	544 (37.2%)
Education						
Primary school	1378 (24.3%)	562 (31.1%)	1549 (23.8%)	332 (26.4%)	1378 (21.9%)	398 (27.2%)
Secondary school	3063 (54.1%)	929 (51.5%)	3493 (53.7%)	659 (52.3%)	3397 (54%)	781 (53.5%)
University	1219 (21.5%)	314 (17.4%)	1459 (22.4%)	268 (21.3%)	1516 (24.1%)	282 (19.3%)
Personal income						
Lower quartile			2758 (42.4%)	624 (49.5%)	1571 (24.9%)	417 (28.5%)
Second quartile			2106 (32.4%)	356 (28.2%)	1439 (22.8%)	370 (25.3%)
Third quartile			1208 (18.6%)	205 (16.3%)	1624 (25.8%)	354 (24.2%)
Upper quartile			433 (6.66%)	76 (6.03%)	1668 (26.5%)	322 (22%)
Live alone HUNT1–3						
No	5539 (97.8%)	1599 (88.4%)	6069 (93.3%)	916 (72.6%)	5485 (87%)	749 (51.2%)
Yes	123 (2.17%)	209 (11.6%)	437 (6.72%)	345 (27.4%)	817 (13%)	714 (48.8%)
Low PA	2453 (71.5%)	791 (76.1%)	3391 (80.7%)	678 (83%)	2915 (56.7%)	706 (62%)
High PA	976 (28.5%)	249 (23.9%)	813 (19.3%)	139 (17%)	2226 (43.3%)	433 (38%)
Use of alcohol HUNT1–3						
No excessive	5477 (97.7%)	1741 (97.4%)	5199 (98.2%)	1013 (99.2%)	5957 (95.9%)	1386 (96.9%)
Excessive	129 (2.3%)	47 (2.63%)	95 (1.79%)	8 (.784%)	256 (4.12%)	44 (3.08%)
Obesity HUNT1–3						
No	5299 (93.7%)	1669 (92.5%)	5459 (84.2%)	1032 (82.1%)	4814 (76.6%)	1031 (71.1%)
Yes	358 (6.33%)	136 (7.53%)	1028 (15.8%)	225 (17.9%)	1469 (23.4%)	420 (28.9%)
Had stroke at HUNT1–3						
No			6462 (99.3%)	1247 (98.9%)	6077 (96.4%)	1387 (94.8%)
Yes			44 (.676%)	14 (1.11%)	225 (3.57%)	76 (5.19%)
Smoking HUNT1–3						
Never	2634 (46.7%)	800 (44.6%)	2905 (45%)	539 (43.4%)	2889 (46.9%)	631 (44.4%)
Ever	1647 (29.2%)	472 (26.3%)	2277 (35.3%)	391 (31.5%)	2597 (42.1%)	590 (41.5%)
Current	1365 (24.2%)	520 (29%)	1276 (19.8%)	312 (25.1%)	680 (11%)	201 (14.1%)
Diabetes at HUNT1–3						
No	5637 (99.6%)	1791 (99.1%)	6370 (97.9%)	1227 (97.3%)	5867 (93.1%)	1356 (92.7%)
Yes	24 (.42%)	17 (.94%)	136 (2.09%)	34 (2.7%)	435 (6.9%)	107 (7.31%)

Abbreviations: Hearing TL, hearing threshold level defined by best result at any frequency between 500 and 2000 Hz at the best ear; HUNT, Trøndelag Health Study; PA, physical activity; SD, standard deviation.

**FIGURE 2:**
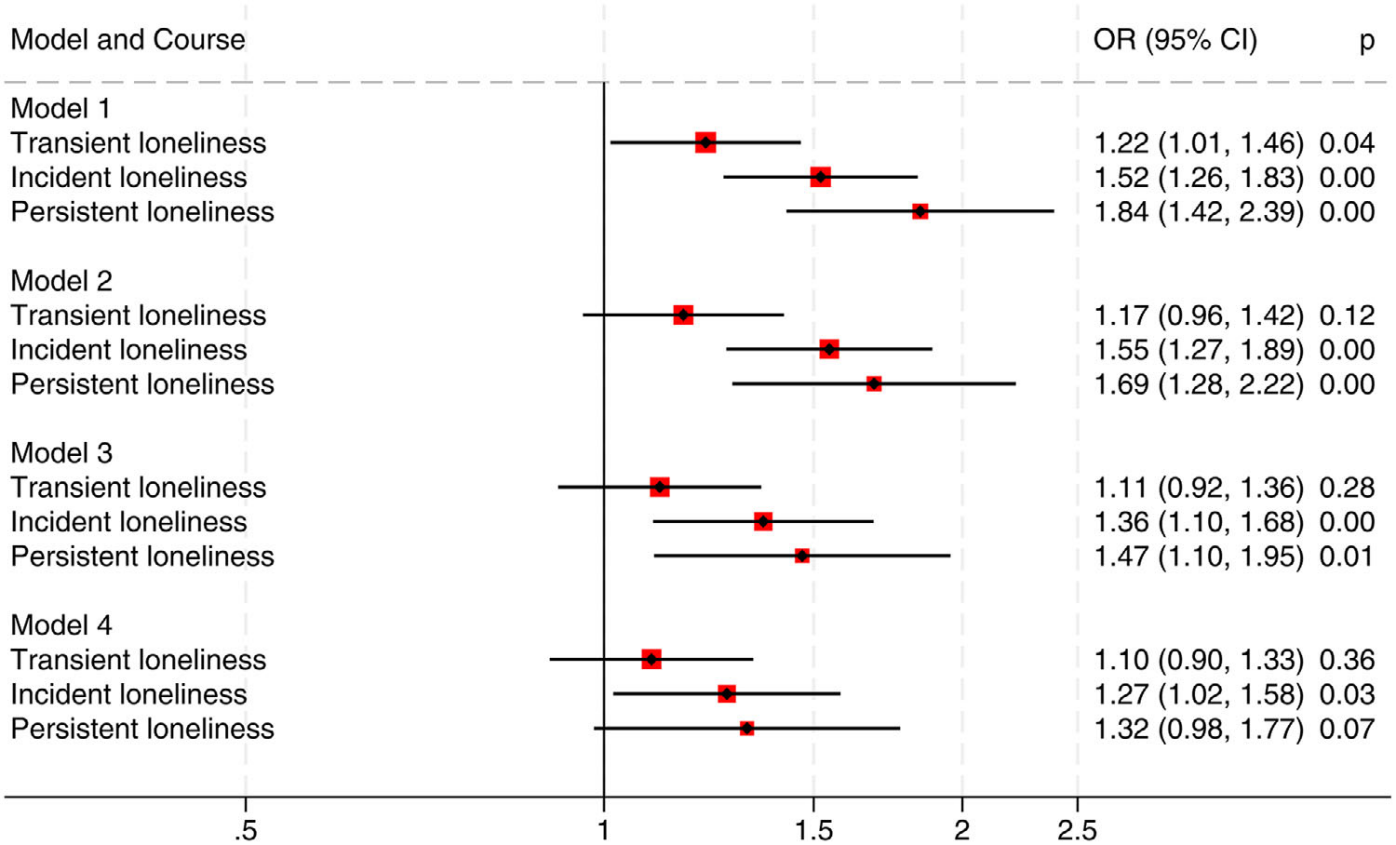
Odds ratio (OR) for dementia by group of loneliness. *N* = 9389. Model 1 is adjusted for age and sex. Model 2 is adjusted for age, sex, education, income, hearing threshold level, frequency of alcohol use, obesity, physical activity, history of smoking, stroke, myocardial infarction, and diabetes. Model 3 is adjusted further for living alone, and Model 4 is adjusted for all the former and depressive symptoms

Participants with persistent loneliness had significantly higher odds of dementia compared to those experiencing no loneliness. In a model adjusted for age and sex (Model 1), the corresponding OR was 1.84 (95% confidence interval [CI] 1.42, 2.39) (Figure 2): after further adjustment in model 2, the odds ratio decreased somewhat (OR 1.69, 95% CI 1.28, 2.22), and when adjusted for living alone in model 3 the odds ratio was 1.47 (95% CI 1.10, 1.95). Participants with incident loneliness also had significantly higher odds of dementia in all three models: Model 1 (OR 1.52, 95% CI 1.26, 1.83), Model 2 (OR 1.55, 95% CI 1.27, 1.89), and Model 3 (OR 1.36, 95% CI 1.10, 1.68). Those who reported transient loneliness did not have higher odds of dementia than those reporting no loneliness in models 2 or 3. In Model 4, which also included adjustment for depression, the results attenuated. For the incident loneliness group OR was 1.27 (95% CI 1.02, 1.58), and for the persistent loneliness group no longer significant, OR 1.32 (95% CI 0.98, 1.77), compared to those with no loneliness (Figure 2).

**FIGURE 3:**
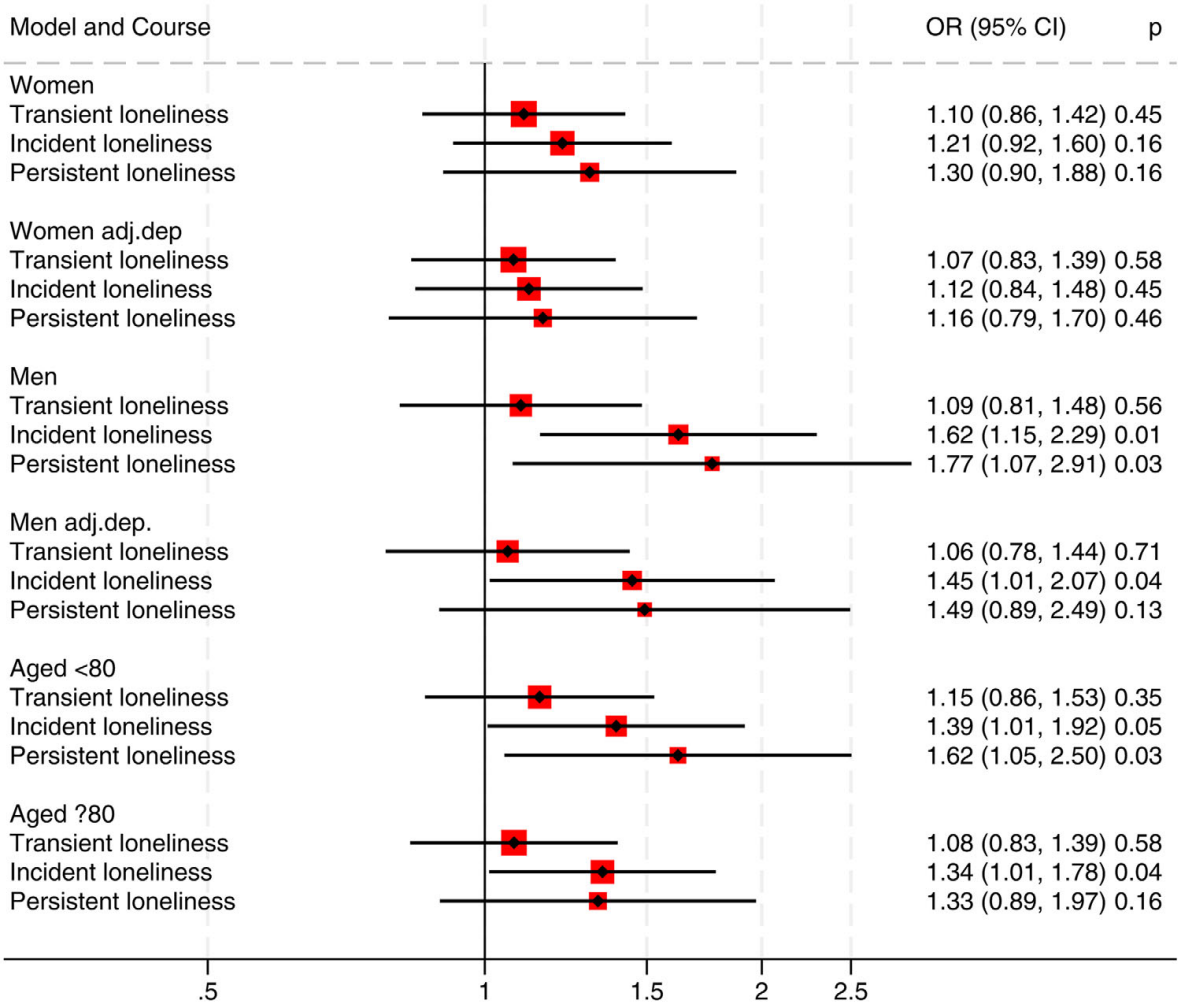
Odds ratio (OR) for dementia at HUNT4 stratified by sex and age for those feeling lonely compared to those not feeling lonely. *n* = 4871 women, *n* = 3896 men, *n* = 2875 aged ≥ 80 years, *n* = 5891. All models are adjusted for age, sex (not in the sex‐stratified analyses), education, income, living alone, hearing threshold level, frequency of alcohol use, obesity, physical activity, history of smoking, stroke, myocardial infarction, and diabetes. Models “Women adj.dep” and “Men adj.dep” denotes that depression is also included in the models. HUNT, Trøndelag Health Study

There was no significant sex*loneliness interaction in relation to dementia risk (Figure 3). In men, the OR for dementia was 1.77 (95% CI 1.07, 2.91) for persistent loneliness versus no loneliness, and 1.62 (95% CI 1.15, 2.29) for incident loneliness versus no loneliness (fully adjusted): in women, the corresponding ORs were 1.30 (95% CI 0.90, 1.88) and 1.21 (95% CI 0.92, 1.60). When adjusting for depression in the sex‐stratified analyses, there was still a significant association remained between incident loneliness and dementia in men (Figure 3). The interaction effect of dichotomized age and course of loneliness was not significant. In stratified analyses, participants younger than 80 years with persistent loneliness had an OR of 1.62 (95% CI 1.05, 2.50) and those with incident loneliness an OR of 1.39 (95% CI 1.01, 2.07) for dementia at HUNT4, compared to those with no loneliness. For those 80 years and older, the corresponding odds ratios were 1.33 (95% CI 0.89, 1.97) and 1.34 (95% CI 1.01, 1.78), respectively (Figure 3).

**FIGURE 4:**
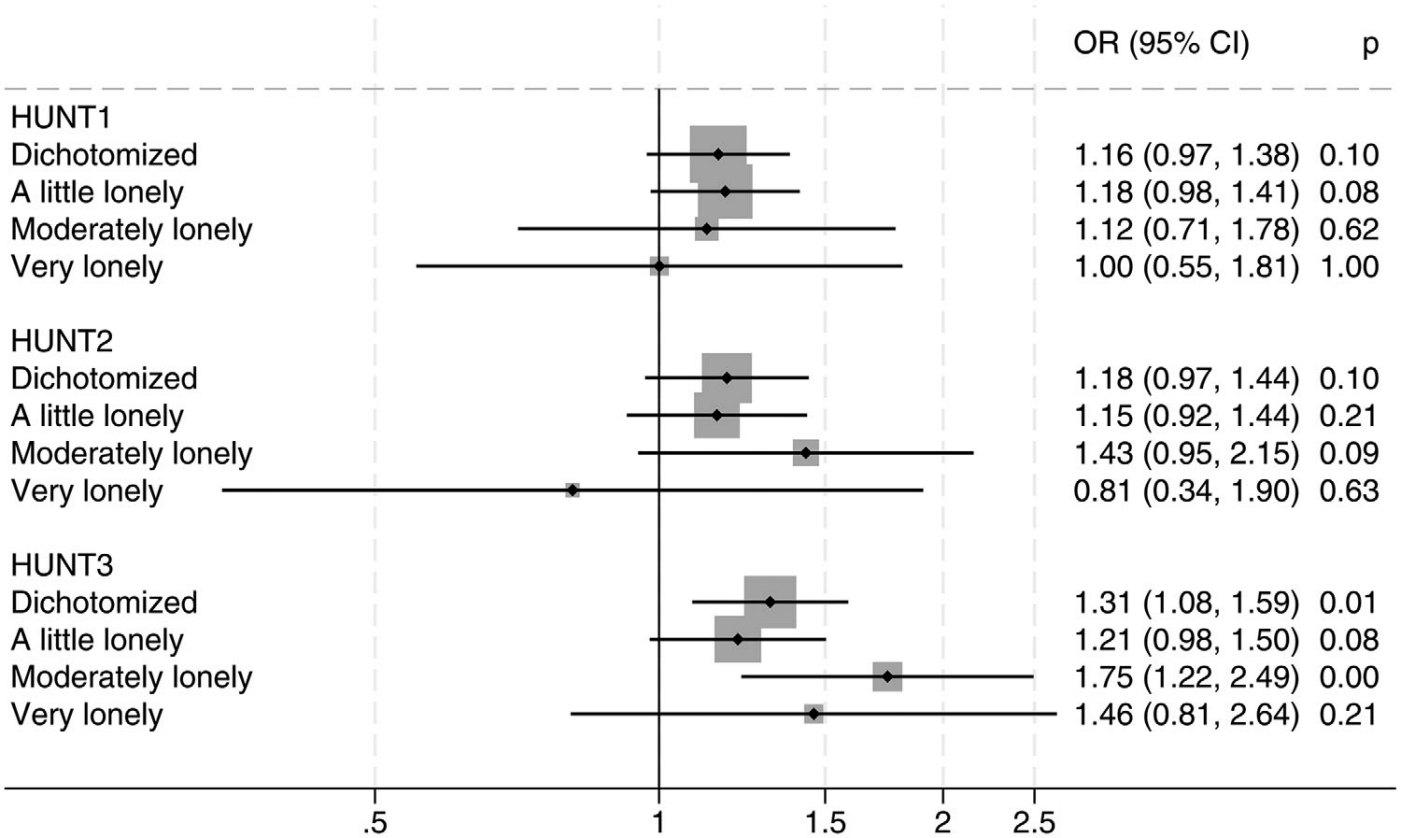
Odds ratio (OR) for dementia at HUNT4 by degree of loneliness at each of the previous HUNT waves. Fully adjusted models, not adjusted for depression. Not lonely as reference. (*n* = 7353 for HUNT1, *n* = 7394 for HUNT2, *n* = 7764 for HUNT3). Note: “Dichotomized” represents the dependent variable loneliness dichotomized into not lonely (ref) and lonely (= a little lonely, moderately lonely, and very lonely). Fully adjusted model is adjusted for age, sex, education, income, living alone, hearing threshold level, frequency of alcohol use, obesity, physical activity, history of smoking, stroke, myocardial infarction, and diabetes. HUNT, Trøndelag Health Study

In the fully adjusted analyses of loneliness measured at each wave, there was an increased odds of later dementia for those lonely at HUNT1 (OR 1.16, 95% CI 0.97, 1.38), HUNT2 (OR 1.18, 95% CI 0.97, 1.44), and HUNT3 (OR 1.31, 95% CI 1.08, 1.59); still, the association was significant only at HUNT3 (Figure 4). The association between the severity of loneliness and dementia did not reveal any clear pattern: the only OR which reached significance was moderate loneliness compared to not being lonely at HUNT3 (OR 1.75 (95% CI 1.22, 2.64)).

### 3.1 Sensitivity analysis

The sensitivity analysis based on complete cases yielded similar results, although the associations between loneliness and dementia were weaker (Table S2).

## 4 DISCUSSION

### 4.1 Course of loneliness

In this cohort study, we examined loneliness—conceptualized as the subjective feeling of being lonely—using repeated measurements over more than three decades and assessed its association with the risk of dementia after age 70. We found that individuals with persistent or incident loneliness had increased odds of developing dementia later in life compared to those who did not experience loneliness. By contrast, individuals with transient loneliness did not exhibit higher odds of later dementia. Incident—but not transient—loneliness may reflect a more recent and possibly enduring disruption in one's social connections during critical later‐life stages when cognitive decline is more likely to manifest. Such a new onset of loneliness might indicate emerging impairments which hinder social engagement, thereby acting as early markers or contributing factors to dementia. In contrast, transient loneliness may represent a temporary or situational experience which does not last long enough to have a significant cumulative effect on cognitive health. Additionally, individuals who experienced transient loneliness may have successfully adapted or re‐established social connections, thereby mitigating the long‐term cognitive impacts. However, in the very early stages of dementia, individuals may feel ashamed or afraid of making errors, leading them to withdraw from social activities and, in turn experience loneliness. These findings highlight the importance of the timing and the duration of loneliness in its relationship with dementia risk.

The increased odds of dementia among individuals experiencing persistent or incident loneliness is supported by a comparable study conducted using data from the Framingham Heart Study.^13^ Another study, involving five repeated measurements of loneliness in the ELSA study found that the long‐term‐high trajectory of loneliness had the strongest association with dementia, and also significant association for long‐term‐moderate and persistently‐increasing loneliness compared to long‐term‐low loneliness.^15^ Furthermore, recent studies from China^25^ and from Australia,^26^ found that only persistent loneliness increased the risk of cognitive decline.

### 4.2 Time between measurement of loneliness and occurrence of dementia

Our finding of increased odds of dementia in those reporting loneliness compared to those not reporting loneliness was only statistically significant at HUNT3, or 11 years before dementia diagnosis. This aligns a large study from UK Biobank with a follow‐up of up to 15 years which found a hazard ratio of 1.45 in a model similar to our fully adjusted model with OR 1.47, although living alone was not included.^10^ However, two other studies also from the UK Biobank using a different measure of loneliness concluded that social isolation rather than loneliness was a risk factor for dementia.^27^, ^28^ Several other studies with follow‐ups of more than 5 years also found a higher risk of dementia to be associated with loneliness.^11^, ^14^, ^29^, ^30^, ^31^, ^32^, ^33^

Our findings concerning the association between the severity of loneliness and dementia showed no clear pattern, but these results should be interpreted with caution as the groups were small.

In our study, we found that adjusting for living alone somewhat attenuated the association between loneliness and dementia, but the results still suggest that loneliness is a dementia risk factor independent of social isolation. Living alone may also precede loneliness, as it also is a risk factor for loneliness.^34^ In comparable studies loneliness has been measured using a variety of instruments. However, these measures have been found to be highly correlated, and self‐rated loneliness shows strong agreement with informant‐ratings.^35^

Several explanations have been proposed for the association between loneliness and dementia. A potential biological mechanism is that loneliness reduces cognitive resilience to age‐related neurodegenerative changes, making individuals who are lonelier more susceptible to negative cognitive outcomes.^36^ Also, it has been hypothesized that loneliness functions as a biological warning system, designed to ensure that members of social species seek companionship—which is essential for survival, prosperity, and reproduction.^37^ A further theory proposes that loneliness triggers the immune system to prepare for physical injuries and reduces preparedness for viral infections (which is relevant if one is isolated from a social group); however, when sustained, the risk of inflammation‐related conditions increases.^38^ Both these latter theories suggest that loneliness may influence behaviors and affective processes, highlighting factors, such as stress, inflammation, and reduced cognitive stimulation as potential mechanisms linking loneliness to adverse health outcomes.^39^ Along these lines, inflammatory markers are associated with all types of dementia.^40^ The pathophysiology leading from loneliness to dementia may involve chronic stress and the activation of the hypothalamic‐pituitary‐adrenal (HPA) axis.^41^ A recent study analyzing autopsy data from individuals with Alzheimer's disease (AD) or cerebrovascular disease found that loneliness was associated with the presence of microinfarcts; the study suggested that the impact of loneliness may occur through cerebrovascular disease rather than through amyloid and tau pathology, even in AD patients.^42^ Loneliness may also lower engagement in health behaviors, reinforcing lifestyle factors as partial mediators along the causal pathway.^43^ In the years before a dementia diagnosis, subclinical decline in cognitive capacity may manifest in reduced social ability, potentially leading to social withdrawal and, consequently, feelings of loneliness. However, a review concluded that reverse causation is unlikely to fully explain the association found between social engagement and cognitive decline.^44^

No significant interaction was found neither between age and loneliness, nor between sex and loneliness in relation to dementia risk. For an extended discussion, see the Supplementary Material.

### 4.3 Loneliness and depression

The association between loneliness and dementia was attenuated after adjusting for depression. This contrasts with a previous study on trajectories of loneliness and dementia risk, where depression had no impact on the results,^15^ and another study demonstrating that loneliness was a risk factor for dementia independent of depression.^13^ Broadly, several studies have found reduced associations between loneliness and dementia after controlling for depression,^10^, ^27^, ^28^, ^30^, ^31^, ^32^ whereas others have found that depression has no impact^11^, ^17^, ^33^, ^45^; a recent meta‐analysis suggests that loneliness is associated with dementia independent of depressive symptoms.^46^ Different measures of depressive symptoms are used, and the prevalence of depression in the study populations varies from 8%^16^ to 25.6%.^18^ Interpreting the role of depression is difficult, as it might act as both a confounding and mediating factor.^15^, ^30^, ^47^

### 4.4 Strengths and limitations

Our study has several strengths, including a large sample size, repeated measurements over three decades, and a robust assessment method for diagnosing dementia. The sample was population‐based, and at HUNT4, assessments were offered at the participants’ homes and in nursing homes to increase inclusion.

Our study also has some limitations. Loneliness was self‐reported through a single question. As a subjective experience, it is appropriate for loneliness to be self‐defined, but its meaning may vary across studies. In addition, the long intervals (> 10 years) between the measurements of loneliness reduces the opportunity to establish more detailed trajectories Furthermore, nearly half of the persons above 70 years who were invited to participate did not do so, and many values were missing for specific covariates, with a high proportion of missing data for the loneliness variable (17.3%–20.4%). Those who did not participate or had missing values might differ from the participants in terms of the prevalence of dementia and/or loneliness, potentially introducing selection bias. Some of this bias was handled by using multiple imputation (i.e., MICE). Additionally, the HUNT population has slightly lower education levels than the general population as there were no large cities in the area, and there are few immigrants compared to the overall Norwegian population; thus, the findings might not be representative of individuals from other racial or cultural backgrounds. However, the association between loneliness and dementia have also been confirmed in diverse populations and settings, including, for example Chinese cohorts^48^ and individuals of various ethnicities in the United States.^30^ One challenge when adjusting for confounders is that some factors might act as both confounders and mediators. Due to a lack of imaging and biomarker data in the diagnostic process, some misclassifications could have occurred, possibly affecting the observed associations.

## 5 CONCLUSION

Our study supports existing literature indicating that loneliness is an independent risk factor for dementia. Both persistent and incident loneliness were associated with increased dementia risk, especially among men and younger individuals. Differences between men and women should be further investigated. Given that loneliness was assessed up to three decades before the dementia assessment, there are substantial opportunities for prevention and intervention strategies focused on reducing loneliness to lower the risk of dementia.

## CONFLICT OF INTEREST STATEMENT

The authors declare no conflict of interest. Authors' disclosure are available in the Supporting Information.

## CONSENT STATEMENT

All participants gave written informed consent.

## Supporting information



Supporting Information

Supporting Information
