# Supplementary material for: Loneliness trajectories and dementia risk: Insights from the HUNT cohort study
Source: Alzheimers Dement (Amst). 2025 Jul 29;17(3):e70154. doi: 10.1002/dad2.70154 (PMC12305116; doi:10.1002/dad2.70154)
Supplement: Supplementary file 1 — Supporting Information [file DAD2-17-e70154-s002.docx]

# SUPPLEMENTARY

## The Trøndelag Health Study (HUNT)

This population-based cohort study was initiated in 1984 when all inhabitants persons aged 20 years and older in the northern Trøndelag region were invited to a comprehensive health examination. Of the invited 88.1%, 75000 persons, participated. About 65 000 participated at the second wave in 1995-1997 (HUNT2), and in the third wave in 2006-2008 (HUNT3) 50 800 aged 20 years and older participated. In the last wave by now 2017-2019 (HUNT4) 56 000 adults participated, and those aged 70 years or older were invited to the extended examination, HUNT4 70+. Each of the four HUNT study waves has been described in detail (1-4), and participants and non-participants have been compared (5, 6)

## Tests used for measurement of cognitive function

**The** **Montreal Cognitive Assessment (MoCA)** is a screening test designed to be quickly performed to detect mild neurocognitive impairment as well as dementia. The tasks include visuospatial, executive, abstraction, memory, language, delayed recall, and orientational aspects. It is found to have high sensitivity and specificity for mild cognitive impairment when the results from MSS-E is in the normal range (7).

**The Word List Memory Task (WLMT)** is a specific test for delayed recall included in the battery use by The Consortium to Establish a Registry for Alzheimer's Disease (CERAD) (8).

**The 8-item Severe Impairment Battery** is a shorter version of the Severe Impairment Battery that can be performed in three minutes instead of twenty. It is designed to measure function to evaluate treatment response in patients with advanced Alzheimer’s disease (9).

## Description of covariates

**Income** was obtained from registry-based information linked from Statistics Norway. Income was grouped in quartiles, based on total (tax included) personal income, from year of participation at HUNT3.

**Education** was obtained from registry-based information linked from Statistics Norway. Education was based on the Norwegian Standard Classification of Education (NUS2000), which is harmonized to the International Standard of Classification of Education (ISCED), and categorized into five levels (unspecified/none, primary school, secondary school, university up to four years, and university more than four years).

**Hearing level** was measured by audiometry at HUNT2, and hearing threshold level (HTL) was defined by the best result at any frequency between 500 and 2000 Hz in the better ear. HTL was used as a continuous variable, and the results from HUNT2 were used in all analyses.

**Cohabiting** was dichotomized into ‘living alone’ and ‘not living alone’, using marital status linked from Statistics Norway for each point in time for HUNT1-3 to supply the self-registration schemes when answers were missing. The self-registration schemes included co-living with parents, children, and other adults or young people.

**Physical activity** was quantified using three questions regarding the intensity, duration, and frequency of physical activities at HUNT1 and HUNT3. At HUNT2, physical activity was quantified by two questions regarding the amount of low-intensity and high-intensity exercise, respectively. HUNT3 included both sets of questions, and other studies have suggested employing a dichotomous measure of physical activity, using a harmonized cutoff either at high (vigorously active vs. not vigorously active) or low (inactive vs. not inactive) levels of physical activity (10, 11). In this study, the ‘low level’ cutoff was used.

**Alcohol consumption** was quantified from self-reported frequency of intake at every HUNT-wave. The questions were not identical in all three waves, and responses were transformed to be comparable: 0 denoted ‘never/few times a year’, 1 was ‘once a month’, 2 was ‘2–3 times a month’, 3 was ‘once a week’, 4 was ‘2–3 times a week’, and 5 was ‘more than 4 times a week’.

**Body weight and height** were measured at participation, using identical protocols at all waves. Body mass index (BMI) was calculated (kg/m2) and dichotomized into no obesity or obesity at cutoff 30kg/m2. Smoking history was categorized as ‘never’, ‘ever’ or ‘currently’.

**Diabetes** was self-reported at all waves and supplied by measurement of non-fasting blood-glucose at HUNT2 and HUNT3, where >11mmol/L was defined as diabetes.

**History of stroke** (yes/no) and ischemic heart attack (yes/no) was self-reported at each wave.

**Depressive symptoms** were measured by the depression subscale (HADS-D) of the Hospital Anxiety and Depression Scale (HADS) at HUNT3. HADS-D consists of seven items, each with four response options on a Likert scale (0-3) yielding a maximum score of 21.

## Extended discussion

### Sex differences

Our results showed no statistically significant sex differences in the association between loneliness and dementia. Further exploration through a stratified analysis revealed no significant association between loneliness and dementia among women, whereas men with incident or persistent loneliness had higher odds of developing dementia. This is in line with a recent study from China, which found that the association between loneliness and dementia was stronger among men than women (12). Men are found to respond to loneliness with a faster decline in most cognitive domains than women (13). It has been suggested that women are not hurt as badly as men by loneliness, probably because they have more multifaceted networks (14). However, other studies have found no difference when testing the interaction between loneliness and sex in relation to dementia risk (15-17), whereas rates of cognitive decline and stress susceptibility may differ between men and women (13, 18). In the analysis stratified by sex, we found that the association between loneliness and dementia was less attenuated by depressive symptoms among men, possibly echoing a study concluding that the impact of loneliness on cognitive resilience tended to be stronger in men than women (14).

References:

1. Holmen J, Midthjell K, Forsen L, Skjerve K, Gorseth M, Oseland A. [A health survey in Nord-Trondelag 1984-86. Participation and comparison of attendants and non-attendants]. Tidsskrift for den Norske laegeforening : tidsskrift for praktisk medicin, ny raekke. 1990;110(15):1973-7.

2. Holmen J, Midthjell K, Krüger Ø, Langhammer A, Holmen TL, Bratberg GH, et al. The Nord-Trøndelag Health Study 1995-97 (HUNT 2): Objectives, contents, methods and participation. Norsk Epidemiologi. 2003;13(1):19-32.

3. Krokstad S, Langhammer A, Hveem K, Holmen TL, Midthjell K, Stene TR, et al. Cohort profile: The HUNT study, Norway. Int J Epidemiol. 2013;42(4):968-77.

4. Åsvold BO, Langhammer A, Rehn TA, Kjelvik G, Grøntvedt TV, Sørgjerd EP, et al. Cohort Profile Update: The HUNT Study, Norway. International journal of epidemiology. 2022.

5. Holmen J, Midthjell K, Forsén L, Skjerve K, Gorseth M, Oseland A. [A health survey in Nord-Trøndelag 1984-86. Participation and comparison of attendants and non-attendants]. Tidsskr Nor Laegeforen. 1990;110(15):1973-7.

6. Langhammer A, Krokstad S, Romundstad P, Heggland J, Holmen J. The HUNT study: participation is associated with survival and depends on socioeconomic status, diseases and symptoms. BMC medical research methodology. 2013;12(1):143.

7. Nasreddine ZS, Phillips NA, Bédirian V, Charbonneau S, Whitehead V, Collin I, et al. The Montreal Cognitive Assessment, MoCA: a brief screening tool for mild cognitive impairment. J Am Geriatr Soc. 2005;53(4):695-9.

8. Morris JC, Heyman A, Mohs RC, Hughes JP, van Belle G, Fillenbaum G, et al. The Consortium to Establish a Registry for Alzheimer's Disease (CERAD). Part I. Clinical and neuropsychological assessment of Alzheimer's disease. Neurology. 1989;39(9):1159-65.

9. Schmitt FA, Saxton J, Ferris SH, Mackell J, Sun Y. Evaluation of an 8-item Severe Impairment Battery (SIB-8) vs. the full SIB in moderate to severe Alzheimer's disease patients participating in a donepezil study. Int J Clin Pract. 2013;67(10):1050-6.

10. Kurtze N, Rangul V, Hustvedt BE, Flanders WD. Reliability and validity of self-reported physical activity in the Nord-Trondelag Health Study: HUNT 1. ScandJPublic Health. 2008;36(1):52-61.

11. Kurtze N, Rangul V, Hustvedt BE, Flanders WD. Reliability and validity of self-reported physical activity in the Nord-Trondelag Health Study (HUNT 2). EurJEpidemiol. 2007;22(6):379-87.

12. Zhou Z, Wang P, Fang Y. Loneliness and the risk of dementia among older Chinese adults: Gender differences. Aging & mental health. 2018;22(4):519-25.

13. Dabiri S, Mwendwa DT, Campbell A. Psychological and neurobiological mechanisms involved in the relationship between loneliness and cognitive decline in older adults. Brain Behav Immun. 2024;116:10-21.

14. Zebhauser A, Hofmann-Xu L, Baumert J, Häfner S, Lacruz ME, Emeny RT, et al. How much does it hurt to be lonely? Mental and physical differences between older men and women in the KORA-Age Study. International journal of geriatric psychiatry. 2014;29(3):245-52.

15. Luchetti M, Terracciano A, Aschwanden D, Lee JH, Stephan Y, Sutin AR. Loneliness is associated with risk of cognitive impairment in the Survey of Health, Ageing and Retirement in Europe. International journal of geriatric psychiatry. 2020;35(7):794-801.

16. Elovainio M, Lahti J, Pirinen M, Pulkki-Råback L, Malmberg A, Lipsanen J, et al. Association of social isolation, loneliness and genetic risk with incidence of dementia: UK Biobank Cohort Study. BMJ open. 2022;12(2):e053936.

17. Montoliu T, Hidalgo V, Salvador A. The relationship between loneliness and cognition in healthy older men and women: The role of cortisol. Psychoneuroendocrinology. 2019;107:270-9.

18. Bale TL, Epperson CN. Sex differences and stress across the lifespan. Nat Neurosci. 2015;18(10):1413-20.

Figure A1 Directed acyclic graph (DAG)
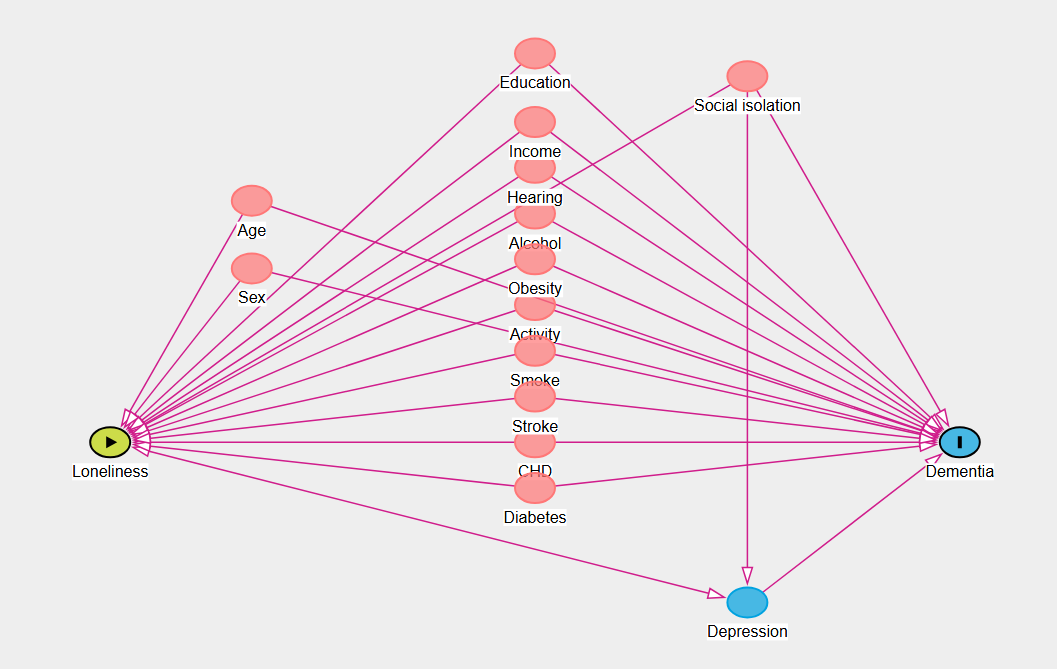


*Abbreviations: Hearing = hearing threshold level, Alcohol = frequency of alcohol intake, Actvity = physical activity, Smoke = smoking history, CHD = coronary heart disease*

**Table A1** Missing observations across HUNT waves in the HUNT Study (Norway), at HUNT1 (1984-86), HUNT2 (1995-97), and HUNT3 (2017-19).

|  | HUNT1 | | | HUNT2 | | | HUNT3 | | |
| --- | --- | --- | --- | --- | --- | --- | --- | --- | --- |
|  | n | missing | % | n | missing | % | n | missing | % |
| participation | 8709 | 680 | 7.2 | 8593 | 796 | 8.5 | 8386 | 1003 | 10.7 |
| lonely | 7471 | 1918 | 20.4 | 7767 | 1622 | 17.3 | 7765 | 1624 | 17.3 |
| living alone | 9358 | 31 | 0.3 | 9365 | 24 | 0.3 | 9380 | 9 | 0.1 |
| Depressive symptoms |  |  |  |  |  |  | 8284 | 1105 | 11.8 |
| alcohol consumption | 7463 | 1926 | 20.5 | 6865 | 2524 | 26.9 | 8131 | 1258 | 13.4 |
| smoking history | 7516 | 1873 | 19.9 | 8507 | 882 | 9.4 | 8108 | 1281 | 13.6 |
| physical activity | 4511 | 4878 | 52.0 | 5321 | 4068 | 43.3 | 6643 | 2746 | 29.2 |
| body mass index | 8645 | 744 | 7.9 | 8567 | 822 | 8.8 | 8353 | 1036 | 11.0 |
| diabetes | 8703 | 686 | 7.3 | 9389 | 0 | 0.0 | 9389 | 0 | 0.0 |
| stroke | 8707 | 682 | 7.3 | 9109 | 280 | 3.0 | 8385 | 1004 | 10.7 |
| heart attack | 8708 | 681 | 7.3 | 9109 | 280 | 3.0 | 9388 | 1 | 0.0 |
| sex |  |  |  |  |  |  | 9389 | 0 | 0.0 |
| education |  |  |  |  |  |  | 9372 | 17 | 0.2 |
| income |  |  |  | 8593 | 796 | 8.5 |  |  |  |
| hearing threshold |  |  |  | 7130 | 2259 | 24.1 |  |  |  |

**Table A2** Odds ratio (OR) for dementia by group of loneliness, complete cases N=3811. No loneliness (ref) n=2470, transient loneliness n=685, incident loneliness n=498, persistent loneliness n=158.

|  | OR | 95% CI | p-value |
| --- | --- | --- | --- |
| Model 1 |  |  |  |
| Lonely-trajectory-group |  |  |  |
| Transient loneliness | 1.26 | (0.96 1.65) | 0.10 |
| Incident loneliness | 1.99 | (1.52 2.60) | <.05 |
| Persistent loneliness | 1.70 | (1.08 2.68) | <.05 |
| Model 2 |  |  |  |
| Lonely-trajectory-group |  |  |  |
| Transient loneliness | 1.11 | (0.83 1.49) | 0.48 |
| Incident loneliness | 1.41 | (1.02 1.94) | <.05 |
| Persistent loneliness | 1.27 | (0.76 2.10) | 0.36 |
| Model 3 |  |  |  |
| Lonely-trajectory-group |  |  |  |
| Transient loneliness | 1.12 | (0.83 1.51) | 0.45 |
| Incident loneliness | 1.39 | (1.00 1.93) | 0.05 |
| Persistent loneliness | 1.15 | (0.68 1.95) | 0.60 |
| Model 4 |  |  |  |
| Lonely-trajectory-group |  |  |  |
| Transient loneliness | 1.08 | (0.80 1.46) | 0.61 |
| Incident loneliness | 1.26 | (0.89 1.77) | 0.19 |
| Persistent loneliness | 1.01 | (0.59 1.74) | 0.96 |
